# Timing and Frequency of Adverse Events Within 90 Days of Hip Arthroplasty

**DOI:** 10.3390/jcm15145635

**Published:** 2026-07-17

**Authors:** Javier Gutiérrez-Adame, Sara Cervera-Bustillo, José Abad-Valle, Paloma Rodríguez-Gómez, Beatriz González-Toledo, Estela Álvarez-Gómez, Carlos Gutiérrez-Ortega

**Affiliations:** 1 Fundación Jiménez Díaz School of Nursing, Autonomous University of Madrid, 28040 Madrid, Spain; jose.abad@quironsalud.es (J.A.-V.); prodriguezg@quironsalud.es (P.R.-G.); beatriz.gonzalezt@fjd.es (B.G.-T.); estela.alvarez@quironsalud.es (E.Á.-G.); 2 Health Research Institute-Fundación Jiménez Díaz University Hospital, Universidad Autónoma de Madrid (IIS-FJD, UAM), 28040 Madrid, Spain; 3 Postsurgical Intensive Care Unit, Hospital Universitario Puerta de Hierro Majadahonda, Calle Joaquín Rodrigo 2, 28222 Majadahonda, Spain; sara.cervera@salud.madrid.org; 4 Department of Preventive Medicine, Hospital Central de la Defensa Gómez Ulla, Glorieta del Ejército 1, 28047 Madrid, Spain; kargut13@gmail.com; 5 Faculty of Medicine and Health Sciences, University of Alcalá, 28801 Alcalá de Henares, Spain

**Keywords:** arthroplasty, replacement, hip, postoperative complications, survival analysis, Kaplan–Meier estimate, patient safety, surgical-wound infection

## Abstract

**Background/Objectives**: Hip arthroplasty is highly effective but carries a measurable burden of early postoperative adverse events. Knowing not only how often these events occur but when they appear is relevant to patient safety and postdischarge surveillance. The aim of this study was to describe the frequency and timing of the main adverse events within 90 days of total or partial hip arthroplasty and to compare onset timing between prosthesis types. **Methods**: This was a retrospective single-center observational cohort study with a longitudinal survival component. A total of 212 consecutive patients who underwent total or partial hip arthroplasty during 2023 at a tertiary university hospital were included. Five adverse events (postoperative fever, surgical-wound infection, prosthesis infection, reoperation, dislocation) were recorded for 90 days from surgery. Time-to-onset was analyzed with the Kaplan–Meier method and compared between total and partial prostheses with the log-rank test (SPSS v25; two-sided *p* < 0.05). **Results**: A total of 167 patients (78.8%) received a total and 45 (21.2%) a partial prosthesis; approximately 29% experienced at least one adverse event. Postoperative fever was the most frequent (58; 27.4%) and earliest event (median onset 2 days, 95% CI 1.5–2.5), followed by surgical-wound infection (17; 8.0%), reoperation (11; 5.2%; median 25 days), prosthesis infection (8; 3.8%; latest onset day 77) and dislocation (5; 2.4%; median 15 days). Onset timing differed between prosthesis types only for fever (median 1 vs. 3 days; *p* = 0.016), an exploratory difference given the different clinical profiles of the two groups. **Conclusions**: Within 90 days of hip arthroplasty, fever is the most frequent and earliest adverse event, whereas wound and prosthesis infections, reoperation and dislocation may present well beyond hospital discharge. These exploratory findings suggest that structured postdischarge follow-up may warrant further evaluation.

## 1. Introduction

Hip arthroplasty is one of the most frequently performed and effective orthopedic procedures; it reliably reduces pain and improves quality of life in patients with advanced hip disease [1,2]. Its volume is high and continues to rise: more than 100,000 total hip replacements are performed annually in the United Kingdom and over 370,000 in the United States, where the figure is projected to exceed 600,000 by 2030 [3,4].

Two main procedures are used. Total hip arthroplasty replaces both the femoral head and the acetabulum, whereas partial arthroplasty (hemiarthroplasty) replaces only the femoral head and is used mainly for displaced femoral neck fractures, preserving more native bone [5,6]. The choice of implant and technique is multifactorial, depending on age, bone quality, indication and cost [7]. Appropriate diagnosis and treatment planning also remain essential in orthopedic surgery, because surgical strategy, complication risk and postoperative outcomes can vary substantially with the underlying pathology and patient characteristics [8].

Despite excellent outcomes, hip arthroplasty is not free of adverse events, and the systematic measurement and monitoring of these events is central to surgical patient safety [9,10]. Surgical-site infection is among the most frequent healthcare-associated infections, typically arising within 30 days of surgery and carrying substantial clinical and economic consequences [11,12]; periprosthetic joint infection specifically affects close to 8% of hip arthroplasty patients in some series [13], with staphylococci among the most common pathogens [14].

The occurrence of adverse events is associated with modifiable and nonmodifiable risk factors, including smoking [15], obesity [16] and diabetes [17], and is closely linked to prolonged hospital stay [18]. Nursing care has an important preventive role: nurse-led intraoperative infection-control routines, adherence to evidence-based practice and infection-prevention link-nurse programs have been shown to reduce adverse events [19,20].

Knowing how often these events occur is not enough on its own; their timing also matters. Because hospital stays after hip arthroplasty are usually short, much of the early window in which complications develop, and in which infections in particular tend to arise within the first weeks after surgery [11,12], falls after the patient has been discharged. Describing when each event tends to appear is therefore relevant for organizing follow-up and detecting complications early.

Although registries have extensively documented long-term implant survival, the frequency, and particularly the timing of the main adverse events during the early postoperative period (especially the first 90 days, when many complications cluster) are less well described, and comparisons of onset timing between total and partial prostheses are scarce. This study therefore set out to describe the frequency and time-to-onset of the main adverse events within 90 days of total or partial hip arthroplasty using the Kaplan–Meier method, and to compare onset timing between prosthesis types.

## 2. Materials and Methods

### 2.1. Study Design and Reporting

A retrospective observational cohort study with a longitudinal survival (time-to-event) component was conducted. The study is reported in accordance with the STROBE statement (Supplementary Materials).

### 2.2. Setting and Participants

The study was conducted at a tertiary care university hospital in Madrid (Spain) that also treats patients referred from other centers. Participants were selected by consecutive non-probabilistic sampling.

Inclusion criteria: patients of both sexes, aged more than 18 years, who underwent total or partial hip arthroplasty during 2023 at the study hospital.

Exclusion criteria: revision of the same hip surgery; patients receiving oncological treatment or who are immunosuppressed; osteomyelitis; avascular necrosis; and hip dysplasia.

### 2.3. Sample Size

The final sample comprised 212 patients. The sample size was estimated with GRANMO v7.12 (Institut Municipal d’Investigació Mèdica, Barcelona, Spain) and is sufficient to estimate the main outcome with a precision of approximately 6% at a 95% confidence level, assuming that about 29% of patients present at least one adverse event.

### 2.4. Variables, Outcomes and Data Sources

The main grouping variable was the type of prosthesis (total vs. partial). Demographic and clinical variables (sex, age, body mass index, smoking, diabetes mellitus, hypertension, dyslipidemia), surgical characteristics (cement use, completion of the surgical checklist, transfusion, ASA class) and laboratory values were collected by reviewing the hospital’s electronic health records.

#### Adverse Events (90-Day Period)

The following events were recorded as present/absent within 90 days of surgery, together with the day of onset: postoperative fever, surgical-wound infection, prosthesis infection, reoperation, and dislocation of the operated hip. “At least one adverse event” was defined as the occurrence of any of the above. Time-to-onset was measured in days from surgery (time origin) to the event, with a maximum observation period of 90 days. Each event was defined a priori by its presence or absence within this period: postoperative fever as a body temperature above 37.5 °C; surgical-wound infection as infection of the surgical wound; prosthesis infection as infection of the implanted hip prosthesis; reoperation as the need for further hip surgery before day 90; and dislocation as dislocation of the operated hip. Because the 37.5 °C threshold for fever also captures the early physiological inflammatory response to surgery, in addition to potential infectious processes, postoperative fever was analyzed as a distinct entity from surgical-wound and prosthesis infection and was interpreted primarily as an early inflammatory marker rather than a confirmed infectious complication.

### 2.5. Statistical Analysis

Patients who did not develop a given event were censored at 90 days. It was verified that no deaths occurred in the sample during follow-up; because such competing events (e.g., death), which could preclude the occurrence of the adverse events under study, were absent, a competing-risks analysis was not required. Comparisons of onset timing between total and partial prostheses were regarded as exploratory, because the two procedures represent different clinical entities and the groups differed at baseline. No multivariable or adjusted survival model was fitted. Between-group comparisons should therefore be read as descriptive and hypothesis-generating rather than as adjusted estimates of the effect of prosthesis type. Continuous variables are summarized as mean (standard deviation) or median (interquartile range) according to distribution (Kolmogorov–Smirnov for *n* ≥ 30, Shapiro–Wilk for *n* < 30); categorical variables as absolute and relative frequencies. Between-group comparisons used the Pearson χ^2^ or Fisher’s exact test for categorical variables, and the Student *t* test or Mann–Whitney U test for continuous variables, as appropriate. For each adverse event, occurrence over the 90-day period was estimated for the whole cohort, and the distribution of time-to-onset among patients who developed the event was estimated with the Kaplan–Meier method; the median time-to-onset with its 95% confidence interval (CI) is reported. Onset timing was compared between total and partial prostheses with the log-rank (Mantel–Cox) test. A two-sided *p* < 0.05 was considered statistically significant. Analyses were performed with SPSS v25.

### 2.6. Ethics

The study was approved by the institutional Research Ethics Committee (CEIm) (approval code EO138-23; favorable opinion dated 24 July 2023, committee meeting of 16 May 2023, minutes no. 09/23) and conducted in accordance with the Declaration of Helsinki and applicable Spanish and EU data-protection legislation. Given the retrospective design and the use of a fully anonymized database, the requirement for individual informed consent was waived by the committee.

### 2.7. Use of Generative Artificial Intelligence

The authors used a generative artificial intelligence tool to assist with the English-language translation and editing of text originally written in Spanish and with document and figure formatting, as detailed in the Acknowledgments. The tool was not used for study design, data collection, statistical analysis, or the interpretation of results. All analyses were performed and verified by the authors, who take full responsibility for the content of the manuscript.

## 3. Results

### 3.1. Cohort Characteristics

A total of 212 patients were included; 99 (46.7%) were men and 113 (53.3%) were women, with a mean age of 69 (SD 13) years and a mean body mass index of 27.2 (SD 4) kg/m^2^. Demographic characteristics and cardiovascular risk factors are shown in Table 1. Of the 212 procedures, 167 (78.8%) were total and 45 (21.2%) were partial hip arthroplasties; surgical characteristics by prosthesis type are shown in Table 2.

### 3.2. Frequency and Timing of Adverse Events

Approximately 29% of patients experienced at least one adverse event within 90 days. Postoperative fever was by far the most frequent event (58 patients; 27.4%), followed by surgical-wound infection (17; 8.0%), reoperation (11; 5.2%), prosthesis infection (8; 3.8%) and dislocation (5; 2.4%) (Table 3). Fever was also the earliest event: more than 40% of cases occurred on the first postoperative day and about 70% by the second day, with a median onset of 2 days (95% CI 1.5–2.5). In contrast, prosthesis infection (first case on day 12, latest on day 77) and reoperation (median 25 days, latest on day 62) frequently presented after the usual hospital discharge.

### 3.3. Comparison Between Prosthesis Types

**Figure 1:**
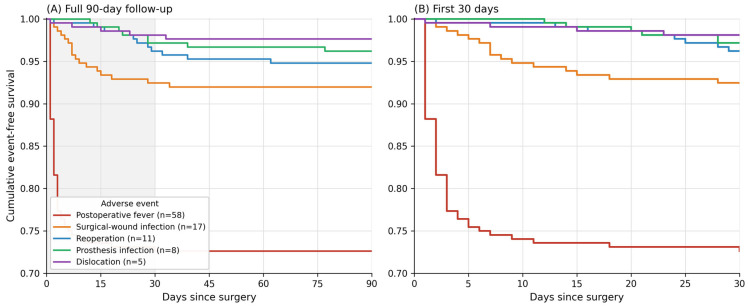
Kaplan–Meier cumulative event-free survival for the five adverse events after total or partial hip arthroplasty (*n* = 212). (**A**) Full 90-day follow-up; the shaded area marks the region magnified in (**B**). (**B**) First 30 days, where most events, including all cases of postoperative fever, occur. The y-axis is truncated at 0.70 to aid visualization; the number of events is shown in the legend.

The only adverse event whose onset timing differed significantly between prosthesis types was postoperative fever, which appeared earlier after total (median 1 day) than after partial arthroplasty (median 3 days; log-rank *p* = 0.016) (Figure 1). For surgical-wound infection (*p* = 0.901), prosthesis infection (*p* = 0.408), reoperation (*p* = 0.491) and dislocation (*p* = 0.754), differences in onset timing were not statistically significant (Table 4). Most prosthesis infections (six of eight), reoperations (nine of 11) and dislocations (four of five) occurred in patients with a total prosthesis, consistent with the larger proportion of total arthroplasties in the cohort.

## 4. Discussion

In this single-center cohort of 212 hip arthroplasties, the most frequent adverse events within 90 days were postoperative fever, surgical-wound infection, reoperation, prosthesis infection and dislocation, events that are consistently reported as the most relevant after hip arthroplasty [21,22,23]. Beyond their frequency, describing when they appear is clinically useful, since several of these complications occur after the usual hospital stay.

**Postoperative fever.** Fever was the most frequent event, affecting about one in four patients (temperature > 37.5 °C over 90 days), and was the earliest to appear (median 2 days). Lower frequencies (around 15%) reported elsewhere may reflect a higher temperature threshold (38 °C) [24,25]. Although fever may signal an early infectious process, it is frequently a self-limiting inflammatory response mediated by cytokines released after surgery [26,27,28]. Onset timing differed by prosthesis type, with a median of 1 day after total versus 3 days after partial arthroplasty (*p* = 0.016); the later onset after partial procedures is consistent with reports that delayed surgery in fracture patients is associated with adverse events and a longer hospital stay [29]. However, because the 37.5 °C threshold largely captures the early physiological inflammatory response rather than infection, and because total and partial arthroplasty correspond to different clinical populations (elective surgery versus hip-fracture repair), this between-group difference in fever timing is of limited clinical relevance and should be regarded as exploratory rather than as evidence of an effect of prosthesis type.

**Surgical-wound infection.** Wound infection occurred in 8% of patients, within the 3–8% range reported in the literature [30,31], and presented at a median of about one week, with roughly half of cases by day 7 and three quarters by two weeks, consistent with previous time-to-onset estimates [32]. Onset timing did not differ significantly between prosthesis types. Smoking is a recognized, modifiable risk factor for this event [33]. Completion of the surgical safety checklist is also relevant to its prevention: although some studies report no effect [34], others describe reductions in infection rates with proper checklist use [31,35,36], which reflects the patient-safety role of the perioperative, frequently nurse-led, team. Wound infection has also been linked to allogeneic transfusion [37] and to a longer hospital stay [38].

**Prosthesis infection.** Periprosthetic joint infection occurred in fewer than 4% of patients, slightly above the 1–3% reported in several cohorts [39,40]. Obesity and diabetes are recognized risk factors for this complication [41], as are cardiovascular conditions such as hypertension [42]. In terms of timing, half of the infections appeared within the first three weeks and three quarters within the first month, with the latest case at day 77; comparable 90-day windows have been described, although data on the day of onset are scarce [43].

**Reoperation and dislocation.** The reoperation rate was around 5%, slightly above the 3–4% reported elsewhere, with dislocation and prosthesis infection being the main reasons for returning to the operating room [44,45]. Dislocation occurred in 2% of patients, closely matching the 1.5–2% reported in the literature [46,47]. For both events the number of cases was small, which limits the precision of the timing estimates.

**Patient-safety implications.** The clustering of fever in the first 48 h is consistent with an early inflammatory postoperative response, whereas the later onset of infections, reoperation and dislocation shows that a substantial share of clinically relevant adverse events appears after discharge. These observations suggest that structured postdischarge follow-up may warrant further evaluation for the early detection and management of complications after hip arthroplasty; however, the present study did not assess any specific surveillance strategy, readmission, or cost-effectiveness, so this remains a hypothesis to be tested. Such follow-up might, for example, concentrate clinical and wound review and selected laboratory testing around the periods when these events tend to appear, potentially supported by telemedicine, although the optimal schedule and its benefit require dedicated prospective studies.

**Clinical applications and future research.** Because this study describes when the main adverse events tend to appear, its most direct application is practical timing. Knowing that fever occurs very early, whereas wound and prosthesis infections, reoperation and dislocation tend to appear later and often after discharge, can help clinicians concentrate clinical review around the periods when each event is most likely, both during admission and after the patient goes home. These are, however, observations from a descriptive, single-center study, and they should be confirmed before being translated into practice. Future work could test whether follow-up timed to these higher-risk periods improves the early detection of complications, and whether the exploratory difference in fever timing between prosthesis types is confirmed in larger samples. Prospective and ideally multicenter studies would also provide the greater number of events needed to estimate the timing of the rarer outcomes, such as prosthesis infection and dislocation, with adequate precision.

**Strengths and limitations.** Strengths include a consecutive cohort with patient-level clinical detail not available in registries, a clearly defined 90-day window, STROBE-compliant reporting, and a single-center design with uniform surgical protocols, team and record-keeping, which reduces inter-center variability and supports the internal consistency of the time-to-onset estimates. Limitations include the retrospective design and the single-center setting, which limits external generalizability; the 90-day window, which cannot capture late complications such as aseptic loosening; the small number of events for the rarer outcomes (prosthesis infection, dislocation), which reduces precision and statistical power; and the descriptive, unadjusted nature of the Kaplan–Meier analysis. Adjusted or multivariable survival modeling was deliberately not performed, given the small number of events and the fact that total and partial arthroplasty represent different clinical populations; the between-group comparisons are therefore exploratory rather than adjusted effect estimates.

## 5. Conclusions

Within 90 days of total or partial hip arthroplasty, postoperative fever is the most frequent and earliest adverse event, whereas wound and prosthesis infections, reoperation and dislocation may present after hospital discharge. The timing of onset differed between prosthesis types only for fever, an exploratory difference of limited clinical relevance. These exploratory findings suggest that structured postdischarge follow-up may warrant further evaluation to detect and manage adverse events after hip arthroplasty, a hypothesis that should be confirmed in prospective, multicenter studies.

## Figures and Tables

**Table 1:** Demographic characteristics and cardiovascular risk factors of the cohort.

Variable	Total (*n* = 212)	Men (*n* = 99)	Women (*n* = 113)
Age, years—mean (SD)	69 (13)	66 (13)	71 (12)
BMI, kg/m^2^—mean (SD)	27.2 (4)	27.3 (4)	27.1 (5)
Smoking—n (%)	30 (14.2)	17 (17.2)	13 (11.5)
Diabetes mellitus—n (%)	24 (11.3)	12 (12.1)	12 (10.6)
Hypertension—n (%)	101 (47.6)	52 (52.5)	49 (43.4)
Dyslipidemia—n (%)	76 (35.8)	37 (37.4)	39 (34.5)

SD, standard deviation; BMI, body mass index. *p* values (vs. sex): age 0.014; BMI 0.616; smoking 0.238; diabetes 0.731; hypertension 0.183; dyslipidemia 0.665.

**Table 2:** Surgical characteristics by prosthesis type.

Characteristic	Overall (*n* = 212)	Total (*n* = 167)	Partial (*n* = 45)	*p*
Female sex—n (%)	113 (53.3)	84 (50.3)	29 (64.4)	0.091
Cement use—n (%)	45 (21.2)	23 (13.8)	22 (48.9)	<0.001
Checklist completed—n (%)	170 (80.2)	162 (97.0)	8 (17.8)	<0.001
Transfusion—n (%)	26 (12.3)	6 (3.6)	20 (45.5)	<0.001
ASA I—n (%)	12 (5.7)	11 (6.6)	1 (2.2)	<0.001
ASA II—n (%)	155 (73.1)	136 (81.4)	19 (42.2)	
ASA III—n (%)	43 (20.3)	19 (11.4)	24 (53.3)	
ASA IV—n (%)	2 (0.9)	1 (0.6)	1 (2.2)	

ASA, American Society of Anesthesiologists physical status. The single *p* value refers to the overall ASA distribution.

**Table 3:** Frequency and onset window of adverse events within 90 days, overall and by prosthesis type.

Adverse Event	Overall *n* (%)	Total (*n*)	Partial (*n*)	Onset, Day (First–Last)
Postoperative fever	58 (27.4)	41	17	1–30
Surgical-wound infection	17 (8.0)	9	8	2–34
Reoperation	11 (5.2)	9	2	2–62
Prosthesis infection	8 (3.8)	6	2	12–77
Dislocation	5 (2.4)	4	1	1–33
≥1 adverse event	≈29%	—	—	—

**Table 4:** Kaplan–Meier median time-to-onset (days) by prosthesis type and log-rank comparison.

Adverse Event	Total—Median (95% CI)	Partial—Median (95% CI)	Log-Rank *p*
Postoperative fever	1 (—)	3 (1.57–7.97)	0.016
Surgical-wound infection	9 (6.08–11.92)	7 (6.20–7.80)	0.901
Prosthesis infection	21 (14.60–27.40)	14 (—)	0.408
Reoperation	28 (7.55–48.45)	24 (—)	0.491
Dislocation	7 (0–20.72)	23 (—)	0.754

CI, confidence interval. “—“ = CI not estimable/not reported.

## Data Availability

The anonymized data are available from the corresponding author upon reasonable request, subject to ethical and legal restrictions.

## Supplementary Materials

The following supporting information can be downloaded at: https://www.mdpi.com/article/10.3390/jcm15145635/s1, Figure S1: STROBE flow diagram; STROBE Statement: checklist of items that should be included in reports of observational studies.

## Abbreviations

The following abbreviations are used in this manuscript:

ASA	American Society of Anesthesiologists (physical status classification)
BMI	body mass index
CEIm	Research Ethics Committee (Comité de Ética de la Investigación con medicamentos)
CI	confidence interval
SD	standard deviation
STROBE	Strengthening the Reporting of Observational Studies in Epidemiology
