# Supplementary material for: Timing and Frequency of Adverse Events Within 90 Days of Hip Arthroplasty
Source: J Clin Med. 2026 Jul 17;15(14):5635. doi: 10.3390/jcm15145635 (PMC13412914; doi:10.3390/jcm15145635)
Supplement: Supplementary file 1 [file jcm-15-05635-s001.zip › STROBE flow diagram.pdf]

## STROBE flow diagram

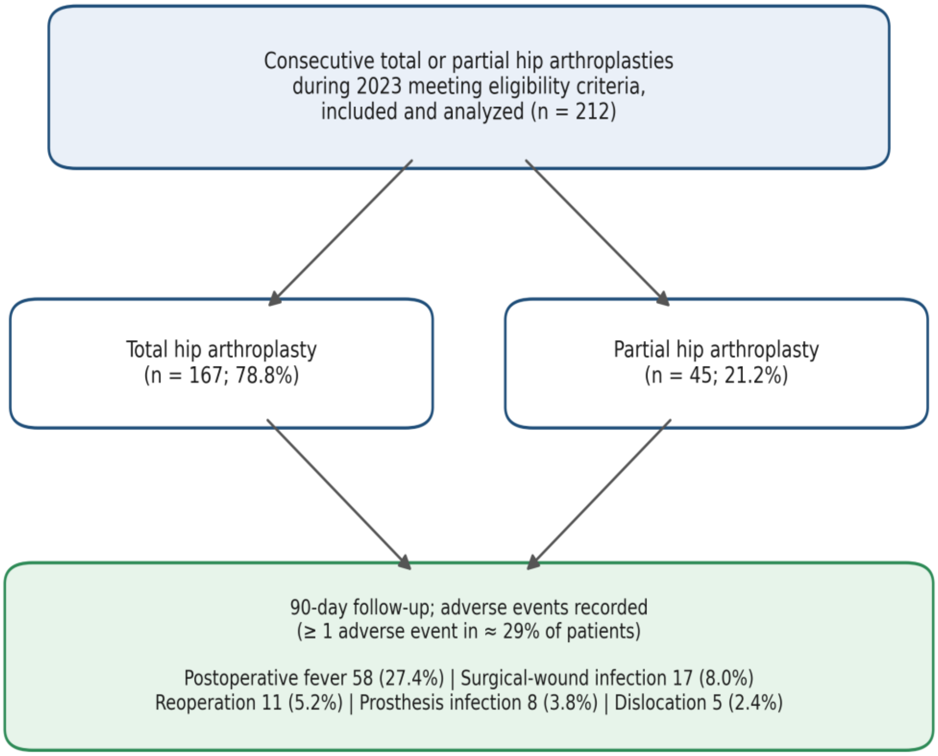


**Figure S1.** Flow of participants. All consecutive patients undergoing total or partial hip arthroplasty during 2023 who met the eligibility criteria were included and analyzed (n = 212; 167 total and 45 partial prostheses) and followed for 90 days for the occurrence of adverse events. The following exclusion criteria were applied a priori: revision of the same hip surgery; oncological treatment or immunosuppression; osteomyelitis; avascular necrosis; and hip dysplasia. As the study used consecutive sampling and a formal screening log was not maintained, the number of patients assessed and excluded by each criterion is not available.
